# Higher Body Mass Index Is Associated with Subjective Olfactory Dysfunction

**DOI:** 10.1155/2015/675635

**Published:** 2015-06-25

**Authors:** Z. M. Patel, J. M. DelGaudio, S. K. Wise

**Affiliations:** Department of Otolaryngology, Emory University School of Medicine, Atlanta, GA 30308, USA

## Abstract

*Background*. Morbidly obese patients demonstrate altered olfactory acuity. There has been no study directly assessing Body Mass Index (BMI) in patients with olfactory dysfunction. Our purpose was to compare BMI in a group of patients with subjective olfactory dysfunction to those without subjective olfactory complaints. *Methods*. Retrospective matched case-control study. Sixty patients who presented to a tertiary care otolaryngology center with subjective smell dysfunction over one year were identified. Neoplastic and obstructive etiologies were excluded. Demographics, BMI, and smoking status were reviewed. Sixty age, gender, and race matched control patients were selected for comparison. Chi-square testing was used. *Results*. 48 out of 60 patients (80%) in the olfactory dysfunction group fell into the overweight or obese categories, compared to 36 out of 60 patients (60%) in the control group. There was a statistically significant difference between the olfactory dysfunction and control groups for this stratified BMI (*p* = 0.0168).  *Conclusion*. This study suggests high BMI is associated with olfactory dysfunction. Prospective clinical research should examine this further to determine if increasing BMI may be a risk factor in olfactory loss and to elucidate what role olfactory loss may play in diet and feeding habits of obese patients.

## 1. Introduction

The epidemic of obesity has been well described, with close to 65% of the adult population categorized as overweight or obese, showing an increase of 48% and 134%, respectively, since the early 1980s. At this time approximately 1 in 20 Americans have a Body Mass Index (BMI) >40 kg/m^2^ [1]. Obesity has been shown to cause a variety of diseases, is linked to almost 300,000 deaths each year, and is responsible for $117 billion in direct and indirect costs annually in the United States [2].

Olfactory dysfunction also affects a significant portion of the population, with 5% of the general population being anosmic and 15% considered to be hyposmic [3, 4]. Patients with hyposmia have a decreased ability to perceive smell, while those with anosmia have the inability to perceive a smell. The prevalence of smell dysfunction increases with age, with hyposmia being identified in approximately 20 to 25% of patients over the age of 50 [1, 5–7].

The sense of smell plays a central role in quality of life and environmental awareness. Because olfactory input contributes up to 80% of the flavor of our food, smell loss can greatly modify the sense of taste and the associated enjoyment of that food, resulting in altered feeding habits and appetite [2, 8, 9]. The sense of smell is also an important guard against dangerous stimuli such as spoiled food, toxins, smoke, or natural gas leakage [1, 2, 7].

As food and drink are such a central part of human culture and interaction, regardless of geographic location or socioeconomic status, olfactory disorders have been associated with social isolation, depression, and mood changes [10–12]. Thus, olfactory loss adversely affects patients' quality of life, health, and safety.

Common causes of olfactory loss can be divided into two main classes: conductive or sensorineural losses. These include such broad ranging etiologies as nasal polyps, chronic rhinosinusitis (CRS), upper respiratory infections (URI), traumatic injury, and neurodegenerative diseases [1, 2, 6, 7]. Other known causes include toxin exposure, endocrine or hormonal abnormalities, iatrogenic loss, tumors, age-related loss, and a myriad of others [1, 2, 11, 13]. In many patients, we are unable to identify an etiology.

Multiple studies have shown that odor acquires the ability to modify both preparatory and satiety-related components of ingestion, and thus olfaction may play a large role in the development of obesity and the resistance we see in these patients to weight loss methodologies [14]. Animal models indicate that fasting increases and satiation decreases olfactory detection [15], and recently it was shown that the peptide hormone adiponectin, considered to be a starvation signal, increases the responsiveness of the olfactory system [16]. A decade ago, it was shown that morbidly obese individuals (BMI > 45) were more likely to show olfactory dysfunction than moderately obese individuals [17], but it has never been shown if olfactory function is related to BMI in any other group. The purpose of our study was to compare BMI in a group of patients with subjective olfactory complaints with the BMI of patients without subjective olfactory complaints.

## 2. Materials and Methods

A retrospective case-control chart review was performed after obtaining approval from the Emory University Institutional Review Board. Patients presenting to the Emory Otolaryngology Department over the course of one year (3/2013-3/2014) with subjective olfactory dysfunction were identified using International Classification of Disease, Ninth Revision (ICD-9) diagnosis codes. Patients with either obstructive or neoplastic etiologies of smell disturbance were excluded from this review, using endoscopy and imaging, as were patients who had not suffered from olfactory dysfunction for at least 3 months. Patients with neurodegenerative disorders (such as Alzheimer's and Parkinson's disease) were also excluded. Sixty patients were identified as meeting these criteria who had hyposmia or anosmia indicated both by diagnosis code (781.1) as well as on a validated patient reported quality of life scale (the Sinonasal Outcomes Test, SNOT-22). Patients were included if they had both the diagnosis code and if they reported #21 (sense of taste and smell) on the SNOT-22 scale as the primary and most important complaint (i.e., a level 5, with no other questions receiving higher response than a level 2).

Demographic data such as age, gender, and race were reviewed. Sixty age, gender, and race matched control patients were selected from those presenting to the otolaryngology practice over the same time period, but without subjective olfactory dysfunction. We then reviewed the charts of all 120 patients to examine BMI. We excluded any patients who reported significant weight loss or gain in the interim between the onset of olfactory dysfunction and the time of the visit. We also reviewed the charts for smoking status as this has been shown to affect smell and taste [1, 2]. Comparison of the two groups was made using Chi-square testing.

## 3. Results

A chart review from 3/2013–3/2014 revealed a group of sixty patients with subjective olfactory dysfunction using the criteria established above. The ages ranged from 19 years to 85 years, with a mean of 58. Race and ethnicity breakdown consisted of 3 Asian, 24 black, 2 Hispanic, and 31 white patients. Gender breakdown consisted of 40 female and 20 male patients (Table 1). Etiology of olfactory dysfunction was found to be inflammatory (nonobstructive rhinitis or sinusitis) in 13 (22%), post-URI in 14 (23%), traumatic in 4 (7%), iatrogenic in 1 (<1%), and idiopathic in 28 (47%). Duration of olfactory loss ranged from 3 to 48 months, with a mean of 15 months.

A careful selection of a control cohort gave us sixty age, gender, and race matched patients that had also been seen in the Otolaryngology Department over the same time period but who did not suffer from olfactory complaints.

**Figure 1:**
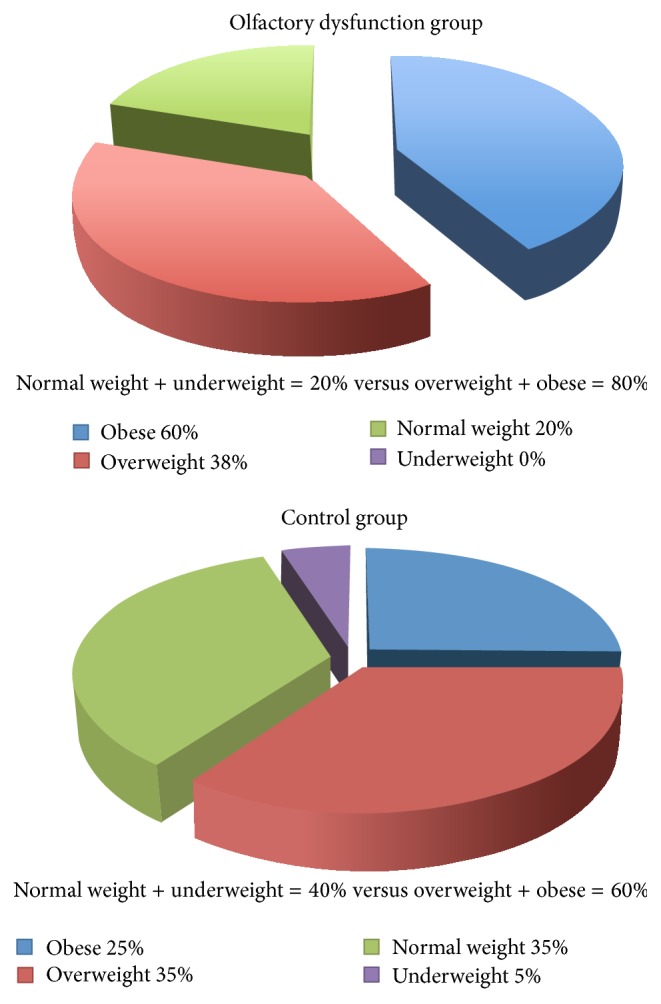
BMI of patients with and without olfactory dysfunction (*p* = 0.0168).

After review of all 120 patients to examine if BMI correlated with normal weight, overweight, or obesity, we found that 48 out of 60 patients (80%) in the olfactory dysfunction group fell into the overweight or obese categories, compared to 36 out of 60 patients (60%) in the control group. Chi-square testing showed this was a statistically significant difference (*p* = 0.0168) (Figure 1). We also reviewed the medical record of all 120 patients to evaluate whether they were or were not smokers to assure we accounted for this possible confounding factor. There was no significant difference between the olfactory dysfunction group and control group in this measure.

## 4. Discussion

As Yeomans stated in 2006, understanding the role of olfactory perception on appetite will contribute to our broader understanding of how sensory qualities of foods may lead to overconsumption [14]. Stevenson et al. established a model for investigating olfactory-based learning in humans by examining how repeated pairing of novel food odors with sweet and sour tastes altered the subsequent experience of the odor presented alone [18]. Interestingly, odors presented in this way acquired the sensory qualities of the paired tastant. For example, when an odor was paired with sucrose, it was then experienced as a sweeter smell by the subject upon subsequent presentation [19]. These experiments established the model through which acquired sensory and hedonic characteristics of food-paired odors could be evaluated further and eventually demonstrated findings of significant value to the assessment of eating habits in obesity.

In line with olfactory receptor neurons showing responsiveness to the starvation signal peptide adiponectin and animal models showing increased olfactory acuity in a starvation or fasting state, human experiments showed that physiologic mechanisms acquired by this association of odor and taste when trained in a hungry state were not expressed when tested in a sated state [20]. The importance of this finding is illuminated by the physiologic mechanism of sensory-specific satiety. This refers to the reduction in pleasantness of a consumed food relative to other foods and appears to play an important role in the normal decision to stop eating [21]. It does not require extensive extrapolation from this basic reflex to suggest that as olfactory dysfunction increases with increasing BMI, the normal olfactory mechanisms that allow humans to control portion size and express satiety are defunct, which contributes to a cycle of increasing food intake and increasing obesity.

Since Buck and Axel uncovered the primary mechanisms of mammalian olfaction in 1991, basic science research has given us an increasingly detailed and complex understanding of the human sense of smell [22]. The breakdown of etiologies in our own group of patients is largely in line with what has been revealed in prior studies, with the smaller percentage of traumatic cases likely due to the smaller number of patients in our study [23–26]. Multiple underlying causes that are either the primary factor or contributing factors to olfactory loss and dysfunction have been identified, including neurologic, metabolic, hematologic, and immunologic pathologies [26]. The multiple and varied metabolic changes that take place in the human body with increasing BMI are the likely culprit for the olfactory dysfunction seen in these patients, but further study is necessary to elucidate mechanisms.

There are biases inherent in any retrospective review and we acknowledge these as a limitation of this type of study. We also acknowledge that a limitation of this study is that the olfactory dysfunction patient group was based on subjective findings, and no validated smell identification tests were performed. In our practice, we have historically reserved objective smell testing for patients with workman's compensation issues or patients suspected of malingering. Unfortunately, even when smell loss is confirmed with these tests, there has been no proven method of helping these patients regain olfactory function. Thus we had not found it beneficial to apply objective smell testing to every patient with subjective olfactory dysfunction. However, as we move to investigate this fascinating association further, validated smell tests will certainly also be used for research purposes.

## 5. Conclusions

High BMI appears to be associated with olfactory dysfunction. Prospective clinical research should examine this further to determine if increasing BMI may be a risk factor for olfactory loss or lack of olfactory recovery after an inciting event and to elucidate what role olfactory loss may play in diet and feeding habits of obese patients.

## Figures and Tables

**Table 1:** Demographic data for patients with or without olfactory dysfunction.

Age (years)	Gender	Race
19–85 (mean 58)	40 female (67%) 20 male (33%)	3 Asian (5%) 24 black (40%) 2 Hispanic (3%) 31 white (52%)
